# Updated Gene Therapy for Renal Inborn Errors of Metabolism

**DOI:** 10.3390/genes16050516

**Published:** 2025-04-29

**Authors:** Sean Hergenrother, Mustafa Husein, Cole Thompson, Ethan Kalina, Rupesh Raina

**Affiliations:** 1 Department of Medicine, Northeast Ohio Medical University, Rootstown, OH 44272, USA; shergenrother@neomed.edu (S.H.); mhusein@neomed.edu (M.H.); cthompson3@neomed.edu (C.T.); ekalina@neomed.edu (E.K.); 2 Akron Nephrology Associates, Cleveland Clinic Akron General Medical Center, Akron, OH 44307, USA; 3 Department of Nephrology, Akron Children’s Hospital, Akron, OH 44308, USA

**Keywords:** inborn errors of metabolism, kidney, primary hyperoxaluria, argininemia, glycogen storage diseases, Fabry disease, adeno-associated virus vectors

## Abstract

Inborn errors of metabolism (IEMs) are a group of disorders resulting from defects in enzymes in metabolic pathways. These disorders impact the processing of metabolites, leading to a wide array of effects on each organ system. Advances in genetic screening have allowed for the early identification and intervention of IEMs, traditionally in the form of enzyme replacement or vitamin supplementation. However, many IEMs disrupt essential metabolic pathways where simple supplementation proves ineffective, resulting in substantial disease burden. In the case of renal IEMs, metabolic pathway disruption leads to the onset of chronic kidney disease (CKD). For these diseases, genetic therapy provides hope. Over the past few decades, the technology for genetic therapy has emerged as a promising solution to these disorders. These therapies aim to correct the source of the defect in the genetic code so that patients may live full, unencumbered lives. In this review, we searched a large database to identify IEMs that affect the kidney and investigated the current landscape and progression of gene therapy technology. Multiple promising genetic therapies were identified for IEMs affecting the kidney, including primary hyperoxaluria, argininemia, glycogen storage diseases Ia and Ib, and Fabry disease. Emerging gene therapy approaches using adeno-associated virus (AAV) vectors, lentiviral vectors, and CRISPR/Cas9 techniques hold promising potential to provide curative treatments for additional single-mutation disorders.

## 1. Introduction

Inborn errors of metabolism (IEMs) are a broad group of rare genetic disorders characterized by an enzyme deficiency in a critical metabolic pathway. While each IEM is rare, with prevalence ranging from 1 in 1000 in phenylketonuria [1] to up to 1 in 117,000 in Fabry disease [2], there is estimated to be over 1000 total IEMs, making their pooled prevalence estimated to be about 1 in 800 live births [3]. These disorders often manifest in the first decade of life and, historically, have had poor long-term prognoses due to a lack of targeted therapies [4]. Treatment varies depending on the primary disturbance but usually entails avoidance of offending carbohydrates, fatty acids, or amino acids and supplementation with other carbohydrates, fatty acids, or amino acids. Recent advancements in identification through genetic testing have allowed for early detection and may allow for earlier intervention. Last year, there were 3900 gene therapy studies approved or occurring worldwide [5]. With the significant number of studies occurring in this rapidly changing landscape, our goal is to determine the progression of therapies for nephrogenic IEMs. While many studies are in vitro currently, there is a possibility for rapid growth and adoption, necessitating the review and cataloging of the progress made. With improved technology and refined techniques, gene therapy may become the standard of care in the coming decades. In our review, we aim to summarize the updated gene therapy treatments for IEMs that affect the kidney. 

## 2. Materials and Methods

Using a large database, the Drug Database for Inborn Errors of Metabolism (DDIEM), nephrogenic IEMs were identified. For each disease, the mutated genes, deficient enzymes, treatments, and phenotypes corrected were reported. DDIEM was jointly developed with researchers from KAUST BORG and the University of Cambridge. The database was last updated in July of 2021. The database pulls drugs using ontology metadata and categorizes experimental treatments along with the phenotypes corrected for each IEM. The website is licensed through a “Creative Commons Attribution 4.0 International License”. 

The DDIEM database reports each IEM with a treatment and a corresponding phenotype corrected (Table 1). To ensure each renal IEM was captured, a list of common phenotypes corrected was assembled, and primary or secondary renal impact was assessed through reviewing the primary literature. The phenotypes corrected included in the search were “metabolic acidosis”, “kidney failure”, “chronic kidney disease”, “lactic acidosis”, “urolithiasis”, “abnormal kidney function”, “proximal tubule dysfunction”, “hypertension”, “proteinuria”, and “hematuria”. After the diseases were identified, the treatments available and phenotypes corrected were tabulated in Table 1, the genes affected and enzyme deficiencies were tabulated in Table 2, and, and the age of onset and diagnostic testing was tabulated in Table 3. The gene therapies were then investigated further by searching the PubMed database to ensure the most updated information was reported. Each disease in which a gene therapy was identified was expanded upon further in the discussion section, outlining the progression of trials, use cases, readiness for human use, and safety precautions. 

## 3. Results

### 3.1. Epidemiology

Inborn errors of metabolism are rare and vary significantly by region, making it difficult to collect comprehensive global data. Neonatal screens may only detect up to 50 out of the 1000s of different diseases. Most IEMs are inherited in an autosomal recessive pattern and may involve complete or partial dysfunction of the affected enzyme [6]. There are estimates for many based on single-country or single-center studies. In a study from the UK, the overall reported prevalence was 1 in 800. The prevalence was further broken down as follows: mitochondrial disorders amounted to about 1 in 4929, lysosomal storage disorders to 1 in 5175, amino acid disorders, excluding phenylketonuria, to 1 in 5354, and organic acid disorders to 1 in 7962 [6]. A multicenter retrospective survey in Italy revealed that the incidence of IEMs was 1 in 2550 live births. This was further categorized as follows: sugar disorders amounted to 1 in 19,552, primary lactic acidemias to 1 in 27,106, organic acidopathies to 1 in 21,000, lysosomal disorders to 1 in 8275, urea cycle defects to 1 in 41,506, and amino acidopathies, excluding phenylketonuria, to 1 in 36,389 [7]. A retrospective study from Austria found that from 1921 to 2021, the median prevalence of IEMs was roughly 1 in 5917 live births [7]. Of note, Austria developed a new screening system in 1966, which resulted in a significantly higher degree of detection of IEMs. Another study from South Korea, through their newborn screening program, detected IEMs at a rate of approximately 1 in 2235 [8]. Classical homocystinuria is estimated to have a prevalence of 1 in 200,000 to 1 in 335,000 in the United States, 1 in 1800 in Qatar, and 1 in 6400 in Norway [8]. The wide range of reported prevalence may be attributed to variations in consanguinity rates, detection sensitivity, quality of intervention, etc. Furthermore, since many studies focus on children, they may under-represent the true prevalence, as some IEMs are not diagnosed until adulthood [6]. A systematic analysis estimated that 3.2% of deaths globally are attributable to IEMs. However, the study suggested that this figure may be under-represented, as it was based on data from high-income countries, with no input from low-income countries [7]. Larger multicenter studies are required to determine disease burden worldwide. 

### 3.2. Disorders of Amino Acid Metabolism

#### 3.2.1. Methylmalonic Aciduria and Homocystinuria

Methylmalonic aciduria and homocystinuria (MMADHC) is caused by mutations in the *MMADHC* gene, impairing the metabolism of vitamin B12 into its active forms, leading to the accumulation of methylmalonic acid and homocysteine. The age of onset for type C is generally at birth or the first few weeks of life, whereas for type D, the disease usually manifests in the first 2 years of life [9,10]. Renal involvement includes tubular dysfunction and tubulointerstitial nephritis progressing to CKD. Treatment involves high-dose hydroxocobalamin, betaine to lower homocysteine, and a protein-restricted diet to manage metabolite buildup, though these approaches mitigate symptoms without correcting the genetic defect. Gene therapy with AAV8 viral vectors in animal models with Isolated Methylmalonic Acidemia has shown increased survival and decreased methylmalonic acid. Ongoing research is exploring the use of genome editing and mRNA therapy [11,12,13].

#### 3.2.2. Adenine Phosphoribosyltransferase Deficiency

Adenine phosphoribosyltransferase (APRT) deficiency is an autosomal recessive disorder caused by mutations in the *APRT* gene, leading to a lack of functional enzymes and resulting in the accumulation of 2,8-dihydroxyadenine (2,8-DHA). APRT deficiency can present at any time in infancy to late adulthood, with the average age of 36 years [14]. Clinically, it presents with recurrent kidney stones, crystalluria, and progressive kidney damage, with some patients developing chronic kidney disease or end-stage renal failure if untreated. Treatment involves allopurinol or febuxostat to inhibit xanthine oxidase and reduce 2,8-DHA production, alongside high fluid intake and a low-purine diet to prevent stone formation and preserve renal function. No genetic therapies are clinically available [15,16].

#### 3.2.3. Lysinuric Protein Intolerance

Lysinuric protein intolerance (LPI) is an autosomal recessive disorder caused by mutations in the *SLC7A7* gene, which encodes the Y+L Amino Acid Transporter 1, impairing the transport of cationic amino acids. LPI generally manifests once an infant is weaned off breast milk and solid foods are introduced to the diet [17]. Though a very rare condition, certain studies indicate a higher prevalence in males with a three-to-one ratio [18]. Clinically, renal involvement includes tubular dysfunction, CKD progressing to ESRD, and rare instances of glomerulosclerosis or interstitial nephritis. Treatment focuses on a low-protein diet to reduce amino acid load, citrulline, phenylbutyric acid, and benzoic acid for hyperammonemia, L-lysine supplementation, and benazepril for renoprotection. While genetic therapies are not yet available, mouse models with SLC7A7 knockouts, mirroring LPI’s clinical features, offer a promising model for developing future gene-based treatments [17,18,19,20].

#### 3.2.4. Argininemia

Argininemia is a rare, autosomal recessive disorder that lacks the production of the final enzyme of the urea cycle, arginase I. The lack of arginase I yields an elevation in arginine instead of conversion to ornithine, causing symptoms including seizures, developmental delay, and spasticity, which typically manifest around age 3 [21,22]. A genetic modifier being studied for treatment is an Aav-based (adeno-associated virus) gene therapy. Currently, gene therapy for argininemia is in the animal trial stage. The most recent studies have focused on both the neurological side effects and arginase expression in the liver. In a study by Lee et al., treated mice showed little to no signs of neurological impairment compared to untreated mice [23]. However, they concluded there was insufficient ornithine production, a continued rise in glutamine levels, and deficient arginase expression. In another trial by Hu et al., gene therapy’s overall effects on the liver were studied more than the neurocognitive effects. Like the former study, they also found a continued rise in glutamine levels and the need for ornithine supplementation in mice with argininemia [24]. The key findings from the article included that only 5% of arginase activity was enough to increase survival time significantly in treated mice compared to controls. After a review of both studies, this gene therapy shows promise, but is not ready for human trials [23,24].

### 3.3. Disorders of Carbohydrate Metabolism

#### 3.3.1. Glycogen Storage Diseases (Ia and Ib)

Glycogen storage disease type Ia is caused by a mutation in the *G6PC* gene, leading to a deficiency in glucose-6-phosphatase, which impairs glycogenolysis and gluconeogenesis [25]. Clinically, it manifests with hypoglycemia, lactic acidemia, neutropenia, hyperlipidemia, hepatomegaly, hyperuricemia, nephromegaly due to fat accumulation leading to glomerular disease, proteinuria, and progression to CKD at around 3 to 6 months of age [25,26]. Treatment focuses on frequent meals, cornstarch, ACE inhibitors, and allopurinol [25]. Gene therapy approaches include AAV-based therapies, Ad-mG6Pase and AAV-CG6PGH, and feline immunodeficiency virus vector-based therapy, FIV-hAAT-G6Pase. The administration of these vectors in knockout mice resulted in normalized glucose, cholesterol, and uric acid levels, decreased glycogen deposition in organs, and increased survival [27,28,29]. A 52-week Phase 1 clinical trial evaluating the safety and efficacy of DTX401, an AAV vector-based therapy, demonstrated a favorable safety profile in humans and showed modest improvements in hypoglycemia control and a reduction in the required cornstarch dosage [30].

Glycogen storage disease type Ib is caused by a mutation in the *SLC37A4* gene, resulting in a deficiency of the glucose-6-phosphate transporter [31]. This condition shares a nearly identical renal presentation and treatment approach with GSD Ia. Gene therapy using the AAV therapy, RAAV-GPE-G6PT, in knockout mice effectively normalized blood glucose, reduced glycogen accumulation in the liver and kidneys, prevented neutropenia, and improved renal function, indicating a promising treatment approach [31].

#### 3.3.2. Fructose-1,6-Bisphophate Deficiency

Fructose-1,6-bisphosphatase deficiency is a rare autosomal recessive genetic disorder caused by mutations in the *FBP1* gene, leading to impaired gluconeogenesis [32]. Clinically, it presents with hypoglycemia, lactic acidosis, and ketosis, which is triggered by fasting or fructose intake. Around half of all patients present with an acute crisis within 4 days of life, and all present within 1 year [33]. Renal involvement includes proximal tubular dysfunction, including renal tubular acidosis and aminoaciduria [32]. Treatment focuses on avoiding fasting, limiting fructose intake, and administering D-glucose in acute episodes. Currently, there are no gene therapies available [32,33].

### 3.4. Disorders of Energy Production

#### 3.4.1. Coenzyme Q Deficiency

Coenzyme Q deficiency is a mitochondrial disorder caused by a mutation in at least one of the ten COQ genes. It can result in a range of phenotypes causing neurological issues, kidney disease, and heart problems that present anywhere from birth to late adulthood, with reports of diagnosis in the 70s [34,35]. Early supplementation with high-dose CoQ10 has been shown to improve many symptoms; however, responses can vary based on the mutation and which *COQ* gene was affected [36]. Genetic therapies for CoQ deficiency are currently in the experimental stages. One study used a lentiviral vector to express CoQ9 mRNA and protein in mouse fibroblasts and hematopoietic progenitor cells. The study showed improvement in mitochondrial function, opening up the possibility of using this approach to treat mitochondrial encephalopathies [37].

#### 3.4.2. GRACILE Syndrome

Growth retardation, aminoaciduria, cholestasis, iron overload, lactic acidosis, and early death syndrome (GRACILE syndrome) is a rare mutation of the *BSC1L* gene, seen predominantly in Finnish or consanguineous populations [38]. The *BSC1L* gene codes for a mitochondrial protein, ubiquinol–cytochrome c reductase complex chaperone, which is responsible for the assembly of complex III in the respiratory chain [39]. This mutation presents with a range of phenotypes with varying severity, from twisted hair shafts and sensorineural hearing loss, known as Björnstad syndrome, to Fanconi syndrome and progressive lactic acidosis, which are hallmarks of GRACILE syndrome [40]. About 50% of patients die within the first few days of life, and the remainder die within 4 months, allowing for potential genetic therapy during this window [41]. There have not been any treatments identified for *BSC1L* disorders or GRACILE syndrome, except continuous sodium bicarbonate, in an attempt to quell the lactic acidosis [38]. There is potential for AAV-based gene therapy for GRACILE syndrome, among other rare, single-mutation, mitochondrial disorders, for which there are multiple ongoing clinical trials [42].

#### 3.4.3. Mitochondrial DNA Depletion Syndrome

There are four clinical characterizations of mitochondrial DNA depletion syndromes (MDSs): myopathic, encephalomyopathic, hepatocerebral, and neurogastrointestinal forms [43]. Of these, the encephalomyopathic (types 5 and 7) have the most renal involvement. MDS 5 (*SUCLA2* mutation) leads to a deficiency in succinate-CoA ligase, an enzyme crucial for oxidative phosphorylation [44]. The disease is characterized by a triad of hypotonia, progressive dystonia, and sensorineural deafness that generally presents from birth to early infancy [43,44]. Findings such as increased lactate leading to lactic acidosis, elevated methylmalonic acid, and elevated carnitine further elucidate the diagnosis. There are no gene therapy trials for MDSs; however, there are substrate replacement therapies. Only 20 cases have been reported, and treatment with riboflavin and thiamine can aid in symptom management. However, the prognosis is poor, with most children dying in childhood [43,44]. Gene therapy is likely far away due to the scarcity of these disorders.

### 3.5. Disorders of Organelle Function

#### 3.5.1. Primary Hyperoxaluria

Primary hyperoxaluria is a rare autosomal recessive disorder with the primary presenting symptom being recurrent kidney stones from the overproduction of oxalate. This disease has three types, the most common being Type I, due to mutations in the *AGXT* gene which codes for alanine glyoxylate aminotransferase enzyme (AGT) that yield hyperoxaluria. The average age of symptom onset is between 3 and 4 years old [45]. AGT limits the oxidation of glyoxylate into oxalate, an important step that will predispose patients to multiple kidney stones throughout their lifetime [46]. The currently approved treatments for primary hyperoxaluria include administering fluids, dialysis, and using calcium oxalate crystal inhibitors, like citrate or pyridoxine. The most common gene therapy research is focused on using AAV therapy to incorporate genetic models for the synthesis of AGT in mice [47,48]. These studies showed the effectiveness of gene therapy, with minimal side effects mentioned. Another gene therapy being studied is implementing dicer-substrate small interfering RNAs (DSiRNAs) to inhibit the synthesis of glycolate oxidase. This enzyme is responsible for converting glycolate to glyoxylate, with inhibition (of glycolate oxidase) leading to an overall decrease in urine oxalate levels and a decreased formation of calcium oxalate stones in the urine. Mice were injected with this substrate, and there was a significant decrease in oxalate formation with no adverse events. Although promising, more research must show clear evidence that this treatment can advance to human trials.

#### 3.5.2. Lowe Oculocerebrorenal Syndrome

Lowe syndrome (Oculocerebrorenal Disease) is an X-linked disorder characterized by congenital cataracts, intellectual disability, and kidney dysfunction. It often leads to chronic renal failure, behavioral issues, cataracts, and hypotonia, often present at birth [49,50]. Mutations of the *OCRL* gene cause faulty functioning of the inositol polyphosphate 5-phosphatase (OCRL-1) protein, causing the oculocerebrorenal symptoms visualized in Lowe syndrome. The current gene therapy research aims to utilize Adenine Base Editors (ABEs) to correct premature stop codons associated with the *OCRL* gene. Chen et al. published a recent study utilizing this technology. They used five different ABEs in the hopes of replacing premature stop codons, allowing the correct protein to be produced and the foundation for a “cure” for Lowe syndrome [51]. Once the gene mutation site was identified, ABEs provided bystander mutations to help alter the genetic code to avoid premature stoppage. The study explained the variability in this treatment, with many different factors needing to be controlled for it to work without causing detrimental issues. Due to the observed variability, further research with gene therapy must be continued before any therapy can be used for human trials [51].

#### 3.5.3. Fabry Disease

Fabry disease is due to a lack of α-galactosidase A, causing an accumulation of ceramide trihexoside. The symptoms include peripheral neuropathy, angiokeratomas, progressive renal failure, and cardiovascular disease, which present, on average, at 6 years old in males and 9 years old in females [52,53]. While males exhibit the classic symptoms mentioned above, the symptoms and prevalence are not as well understood in females. This is likely due to inheritance patterns, with females primarily having Fabry disease symptoms secondary to X chromosome inactivation [54]. There are no current publications on gene therapies for treating Fabry disease, but clinical trials are underway. Research by Khan et al. studied Lentivirus-mediated gene transfer into Fabry patient CD34+ cells, with the goal of increasing production of α-galactosidase A [52]. Unfortunately, their study only included five patients, with three of the five electing to discontinue the trial and resume enzyme replacement therapy, the mainstay of treatment at the current time. Another treatment is Isaralgagene civaparvovec (ST-920) gene therapy in adults with Fabry disease. This gene works by upregulating the expression and manufacturing of α-galactosidase A. Reports from Cincinnati Children’s Hospital and Emory School of Medicine both show promise, but we cannot make any conclusions until concrete evidence is provided for review [52].

#### 3.5.4. Congenital Adrenal Hyperplasia

The most common form of Congenital Adrenal Hyperplasia (CAH) is the 21-hydroxylase-deficient subtype. This disease, in the classic form, presents in infancy with an inability to synthesize cortisol, with salt wasting and shunting towards androgen production. In the nonclassical form, a hyperandrogenic state ensues, leading to precocious puberty hirsutism and virilization in female patients at age 13 and masculinization at puberty in genotypically male patients at age 11 [55]. The gene therapies currently studied aim to activate CYP21A2, the enzyme responsible for the synthesis of 21-hydroxylase. Research has been effective by transporting the gene as an AAV vector containing the active gene. In a more recent study, Naiki et al. performed subcutaneous injections of that vector into newborn mice with CAH, followed by periodically checking the levels of both progesterone and deoxycorticosterone, products of 21-hydroxylase in the pathway of adrenal enzyme synthesis [56,57]. Their study used two different injections, with the most effective results stemming from injection into the thigh muscles of mice (instead of specific fibroblast injections). Although the sample sizes were small, their results showed a significant reduction in the progesterone/deoxycorticosterone (DOC) ratio, meaning the treatment was effective in stimulating the production of 21-hydroxylase, yielding the correct conversion of progesterone to DOC [56]. Further research needs to be conducted with this treatment in order for human trials to be considered in the future. 

## 4. Discussion

**Figure 1:**
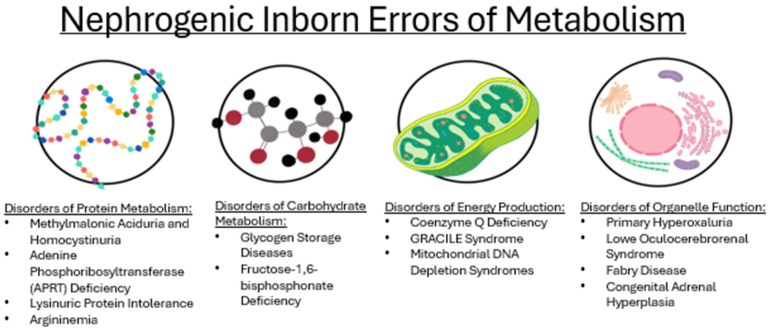
The IEMs that affect the kidney, as listed on the DDIEM database, and their categorization. Graphic created by Lia Hergenrother.

**Figure 2:**
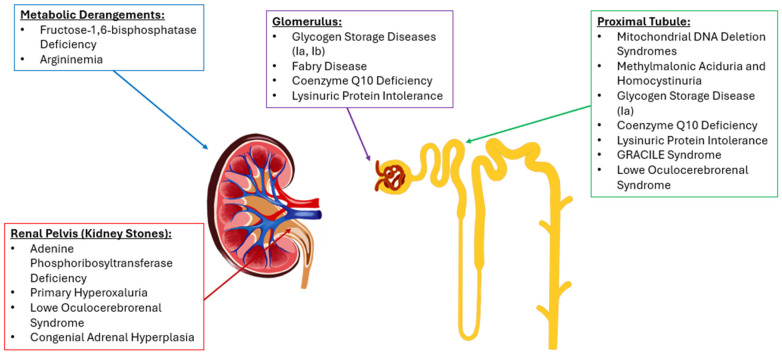
The location along the nephron or in the kidney where each IEM impacts. Graphic created by Lia Hergenrother.

As medical care improves and patients live longer, organ-specific complications, particularly in the kidneys, become increasingly apparent, as the kidneys’ high metabolic demands may make them especially vulnerable to damage in IEMs [58]. Different classifications of IEMs (see Figure 1) impact the kidney through their metabolic derangements. Approximately 10% of known IEMs lead to some renal pathology, affecting all segments of the nephron, including glomerular, tubular, and structural abnormalities (see Figure 2) [58]. For example, Fabry disease affects up to 55% of patients with manifestations like proteinuria, parapelvic cysts, and podocyturia [58]. Similarly, glycogen storage disease type 1 (GSD1) can present with microalbuminuria, hyperfiltration, nephromegaly, and, occasionally, nephrolithiasis due to glycogen accumulation [26]. Fatty acid oxidation defects and a variety of mitochondrial disorders further contribute to renal pathology by causing structural changes, such as kidney cysts and malformations. Moreover, mitochondrial disorders—particularly those affecting coenzyme Q10 biosynthesis—are commonly associated with glomerular pathologies like nephrotic syndrome, while some have even been linked to tubular dysfunction and structural abnormalities, such as kidney hypoplasia and dysplasia [59].

Common renal manifestations that result from IEMs are hemolytic uremic syndrome (HUS), nephrolithiasis, developmental errors, and glomerular damage. Each of these can precipitate kidney function decline and the onset of CKD. HUS, a cause of acute kidney injury, occurs in approximately 10–25% of individuals with combined methylmalonic acidemia and homocystinuria [58]. Methylmalonic acidurias can also lead to tubulointerstitial nephritis and, subsequently, CKD [60]. Many IEMs can present with kidney stone formation, including cystinuria, tyrosinemia, and various forms of primary hyperoxaluria [61]. Disorders of purine and pyrimidine metabolism (e.g., Lesch–Nyhan syndrome, APRT deficiency, xanthinuria) are also commonly linked to stone formation and kidney failure [62,63]. Structural anomalies, such as nephromegaly in GSD1 or renal hypoplasia in Smith–Lemli–Opitz syndrome, highlight the developmental effects of specific IEMs on the kidneys [58]. Lysinuric protein intolerance has been associated with tubulopathy, along with findings of proteinuria and CKD [64]. Despite the availability of supportive therapies, such as enzyme replacement, dietary management, and ACE inhibitors, many IEMs still progress to CKD, emphasizing the limitations of the current treatments [58]. Overall, this highlights the need for a thorough understanding of disease mechanisms across all organs, not just the kidney, to help guide more lasting and targeted therapy approaches to prevent long-term organ damage.

Gene therapy is rapidly transforming the treatment landscape for IEMs, offering promising, targeted approaches to correct underlying genetic defects. The current genetic approaches include adeno-associated virus (AAV) vectors and lentiviral vectors for gene delivery, CRISPR/Cas9 for precise genome editing, RNA-based therapies, such as targeted gene expression or silencing, and tissue-specific strategies that enhance delivery to organs. AAV is a nonpathogenic member of the parvovirus that is engineered to carry therapeutic genes of interest to target cells. The vector is then produced in high titers and injected either systemically into the bloodstream or directly into the target tissue [65,66]. By selecting the appropriate serotypes, these vectors can be tailored to specific tissues [67]. Currently, 13 serotypes and 100 variants have been identified. After administration, the AAV vector attaches to receptors on the cell surface; then, it is taken up into the cell and transported to the nucleus [67,68]. Once inside, the single-stranded DNA genome of the AAV is converted to double-stranded DNA, which is then transcribed and translated to produce the therapeutic protein [68]. AAV therapy has demonstrated success in treating several disorders, including spinal muscular atrophy (SMA), inherited retinal dystrophies, hemophilia, lysosomal storage disorders, aromatic L-amino acid decarboxylase (AADC) deficiency, and more [69]. The advantages of AAV vectors include their ability to provide long-term expression, low immunogenicity, and tissue-specific tropism [70]. AAVs have demonstrated significant potential, with more than 225 clinical trials and six FDA-approved therapies to date [68]. Challenges associated with AAV therapy include the presence of pre-existing antibodies, difficulties in dosing, and manufacturing complexities [68]. AAV vectors are commonly used for liver-directed therapies, such as AAV9 for Pompe disease and AAV8 for phenylketonuria (PKU). AAV8-based gene therapy has shown improved survival in animal models for methylmalonic aciduria and homocystinuria. AAV-based therapies for argininemia have shown improved survival and neurological function in mice, although challenges in arginase expression remain. The neonatal administration of AAV vectors has shown potential for providing long-term correction of metabolic disorders, as seen in mouse models of Maple Syrup Urine Disease using AAV8 vectors [71]. Lentiviral vectors, which integrate into dividing cells, are used in ex vivo hematopoietic stem cell therapies for conditions like CoQ deficiency, MPS I, and metachromatic leukodystrophy [72]. Newer genome-editing technologies, such as CRISPR/Cas9, are being developed to directly correct point mutations in disorders such as tyrosinemia, PKU, and lysosomal storage diseases. RNA-based therapies are also being explored for temporary enzyme replacement in acute metabolic crises [72,73].

One of the more promising therapies for primary hyperoxaluria type I is the dicer-substrate siRNA (DSiRNA), which targets glycolate oxidase. DSiRNA has reduced oxalate formation in animal models, with minimal side effects. Other strategies for gene therapy are tissue-specific, with liver-directed approaches for PKU, OTC deficiency, and glycogen storage diseases and CNS-directed therapies for neuronopathic forms of lysosomal storage disorders, like MPS III. However, not all disorders have viable gene therapies yet—currently, no gene therapies are available for APRT deficiency, Lysinuric Protein Intolerance, or Fructose-1,6-bisphosphatase deficiency. Many are still in the process of developing animal models or remain in early-phase human trials, with limited results reported so far. Clinical trials in this area are virtually nonexistent, and the existing treatments, like enzyme therapy, are often expensive and short-term [74]. As noted earlier, while AAV vectors are widely used for liver-directed therapies, their effectiveness in targeting the kidneys has been limited due to inefficient delivery [75,76]. This is, in part, due to the many different specialized cell types in the kidney [74]. RNA-based therapies use delivery systems, such as lipid nanoparticles, to reach renal cells, while hematopoietic stem cell gene therapy can deliver therapeutic enzymes via differentiated macrophages [73,77]. Challenges remain, including immunogenicity, achieving durable gene expression, manufacturing complexity and cost, and the need for early intervention to prevent irreversible tissue damage. Ongoing research seeks to address these challenges and expand the clinical application of gene therapy.

Some limitations of this review include the omission of certain diseases, which were not categorized by a renal manifestation in the database. This may occur since the database used the exact language in the primary literature cited instead of a standardized language. For example, “lactic acidosis” and “lactic acidemia” were categorized separately in the database despite having the same presentation. As another limitation, due to the scarcity of these disorders, there often is a paucity of large trials clearly outlining disease manifestations, which makes database creation difficult. Also, since a primary function of the kidneys is filtration and excretion of toxins, they are highly susceptible to metabolite disturbances and, therefore, are secondarily affected. This combination results in the renal manifestations of IEMs ambiguous.

## 5. Conclusions

In conclusion, many gene therapies for IEMs are completing clinical trials as the techniques become more sophisticated, efficient, and cost-effective for patients. In this review, we have summarized the updated landscape for genetic therapies for nephrogenic inborn errors of metabolism. The future of gene therapies for single-gene corrections is bright. As our gene editing techniques become more refined, the disease burden may become more manageable, and previously fatal conditions may be cured.

## Figures and Tables

**Table 1:** Disease name, treatment, including gene therapy, and phenotype corrected, as reported in the DDIEM database.

Disease Name	Treatment/Gene Therapy	Phenotype Corrected
Methylmalonic Aciduria and Homocystinuria (Types C and D)	C: Leucovorin + glycine betaine + vitamin B12 D: Vit B12 + glycine betaine + folic acid, protein restriction, carglumic acid	C: Impaired renal function, decreased GFR, elevated total plasma homocysteine, high urine MMA level D: Elevated plasma non-protein-bound homocysteine (hyperhomocystinemia), hypomethioninemia, hyperammonemia
Adenine Phosphoribosyltransferase Deficiency	Allopurinol/Febuxostat	Dysuria, CKD, recurrent UTIs, urolithiasis, hematuria, crystalline nephropathy, elevated urinary 2,8-dihydroxyadenine (DHA) excretion
Lysinuric Protein Intolerance	L-citrulline, Ciclosporin + Amcinonide, phenylbutyric acid, benzoic acid, L-lysine, benazepril	Hyperammonemia, hemophagocytic lymphohistiocytosis, kidney failure, proteinuria, hypertension, orotic aciduria
Argininemia	Benzoic acid, phenylbutyric acid Gene Therapy: Aav-based gene therapy, Aeb1102(pegzilarginase) Co-argi-peg Modified Human Arginase I	Neurotoxicity, hyperammonemia
Glycogen Storage Diseases (Ia and Ib)	I: Alglucosidase Alfa II: High-fat and high-protein diet Gene Therapy: Ia: Dtx401, Fiv-haat-g6pase, Ad-mg6pase, Aav-cg6pgh, Adhd-g6pase Ib: Raav-gpe-g6pt	Hypertrophic cardiomyopathy, mortality II: Elevated CK, weakness 1a, Ib: enlarged kidney, growth failure, lactic acidemia, hyperuricemia
Fructose-1,6-bisphosphatase Deficiency	D-glucose	Hypoglycemia
Coenzyme Q10 Deficiency	Ubidecarenone	Hyperlactatemia, exercise intolerance
GRACILE Syndrome	Sodium bicarbonate	Lactic acidosis
Mitochondrial DNA Depletion Syndromes (4A, 5, 7)	4A: Magnesium, Vatiquinone 5: Vitamin B1 + B2 7: Levocarnitine, glutathione	4A: Seizures 5: Oxidative stress 7: Abnormal circulating creatinine level, oxidative stress
Fabry Disease	Pegunigalsidase Alfa + Agalsidase β, Lucerastat, Migalastat	Abnormal kidney function, high Gb3 accumulation, high mean number of GL-3 inclusions, kidney interstitial capillary
Primary Hyperoxaluria	Pyridoxine + calcium oxalate crystallization inhibitors, pyridoxine, magnesium hydroxide, sodium citrate, potassium citrate, oxalobacter Formigenes Gene Therapy: Aav8-agxt and Aav5-agxt, helper-dependent adenoviral vectors for liver-directed gene therapy, deno-associated virus carrying one copy of Agxt Cdna, polymer-conjugated Agt	Nephrocalcinosis, urolithiasis, abnormal kidney function, high urine oxalate level
Lowe Oculocerebrorenal Syndrome	Sodium bicarbonate, potassium bicarbonate, potassium citrate, Quinethazone, Levocarnitine Gene Therapy: ABE8e-NG-based correction	Proximal tubular dysfunction, high calcium excretion, hypokalemia, metabolic acidosis, secondary carnitine deficiency
Congenital Adrenal Hyperplasia (21-hydroxylase Deficiency)	Hydrocortisone, Dexamethasone Gene Therapy: Aavrh.10-21oh-ha, Adeno-associated viral vector containing Cyp21a1	Hypertension, abnormal circulating renin, aldosterone

GRACILE—growth retardation, aminoaciduria, cholestasis, iron overload, lactic acidosis, and early death, MMA—methylmalonic acid, AAV—adeno-associated virus, Agxt/Agt—alanine–glyoxylate aminotransferase, ABE—adenine base editor.

**Table 2:** Disease name, gene affected, and enzyme deficient for each IEM affecting the kidney. Acquired via the DDIEM database.

Disease Name	Gene Affected	Enzyme Deficient
Methylmalonic aciduria and Homocystinuria (Types C and D)	*MMADHC*	Methylmalonic aciduria and homocystinuria type C/D protein
Adenine Phosphoribosyltransferase Deficiency	*APRT*	Adenine phosphoribosyltransferase
Lysinuric Protein Intolerance	*SLC7A7*	Y+L Amino Acid Transporter 1
Argininemia	*ARG1*	Arginase
Glycogen Storage Diseases (Ia and Ib)	Ia: *G6PC* Ib: *SLC37A4*	I: 7-dehydrocholesterol reductase Ib: Glucose-6-phosphate exchanger SLC37A4 (glucose 6-phosphate translocase)
Fructose-1,6-bisphosphatase Deficiency	*FBP1*	Fructose-1,6-bisphosphatase 1
Coenzyme Q10 Deficiency	*CABC1, ADCK3*	Atypical Kinase COQ8A (mitochondrial)
GRACILE Syndrome	*BCS1L*	Ubiquinol–cytochrome c reductase complex chaperone
Mitochondrial DNA Depletion Syndromes	4A: *POLG* 5: *SUCLA2* 7: *TWNK*	4A: DNA polymerase subunit γ-1 5: Succinyl-CoA synthetase (β subunit) 7: Twinkle mtDNA helicase
Fabry Disease	*GLA*	α-Galactosidase A
Primary Hyperoxaluria	*AGXT*	Serine–pyruvate aminotransferase
Lowe Oculocerebrorenal Syndrome	*OCRL*	Inositol polyphosphate 5-phosphatase OCRL-1
Congenital Adrenal Hyperplasia (21-hydroxylase Deficiency)	*CYP17A1* *CYP21A1*	17-α hydroxylase Frataxin

GRACILE—growth retardation, aminoaciduria, cholestasis, iron overload, lactic acidosis, and early death, mtDNA—mitochondrial DNA.

**Table 3:** Disease name, age of onset, and diagnostic tests used.

Disease Name	Age of Onset	Diagnostic Tests
Methylmalonic aciduria and Homocystinuria (Type C and D)	C: Birth D: <2 years	Plasma Analysis: Elevated methylmalonic acid or homocysteine Genetic Testing: Mutations in *MMADHC* or *MMACHC*
Adenine Phosphoribosyltransferase Deficiency	6 months–72 years (mean = 36.3 years)	Microscopy: 2,8-dihydroxyadenine (DHA) crystals in urine Mass spectrometry: UPLC-MS/MS assay Genetic Testing: Mutations of *APRT* gene
Lysinuric Protein Intolerance	6–12 months (once weaned from breast milk)	Urinalysis: Elevated lysine, ornithine, and arginine in urine Genetic Testing: Mutations in *SLC7A7* gene
Argininemia	3 years	Newborn Screen (NBS) Plasma Analysis: Elevated arginine or ammonia Genetic Testing: Mutations in *ARG1* gene
Glycogen Storage Diseases (Ia and Ib)	3–6 months	Enzyme Assay: Decreased G6Pase (Ia) or G6P-translocase (Ib) activity Genetic Testing: Mutations in *G6PC* (Ia) or *SLC37A4* (Ib) genes
Fructose-1,6-bisphosphatase Deficiency	<1 year (often within first week)	Genetic Testing: Mutations in *FBP1* gene
Coenzyme Q10 Deficiency	Birth–70 years	Muscle Biopsy: Reduced CoQ10 activity Genetic Testing: Mutations in *COQ* genes (*PDSS1, PDSS2, COQ2, COQ4, COQ6, ADCK3, ADCK4, COQ9*)
GRACILE Syndrome	Birth	Genetic Testing: Mutations in *BCS1L* gene
Mitochondrial DNA Depletion Syndromes (4A, 5, 7)	Birth–2 years	PCR: mtDNA deletions Genetic Testing: Mutations in *POLG* (4a), *SUCLA2* (5), and *TWNK* (7) genes
Fabry Disease	Males: Average = 6 years Females: Average = 9 years	Enzyme Assay: Decreased α-galactosidase A Genetic Testing: Mutations in *GLA* gene
Primary Hyperoxaluria	Average = 3–4 years	Urinalysis: Elevated oxalate, glycolate, or glycerate Genetic Testing: Mutations in *AGXT*
Lowe Oculocerebrorenal Syndrome	Birth	Genetic Testing: Mutations in *OCRL* gene
Congenital Adrenal Hyperplasia (21-hydroxylase Deficiency)	Classic: Birth–12 months Nonclassical Males: 11 years Nonclassical Females: 13 years	Plasma Analysis: Elevated 17-hydroxyprogesterone and 21-deoxycortisol

GRACILE—growth retardation, aminoaciduria, cholestasis, iron overload, lactic acidosis, and early death.

## Data Availability

The database can be found at http://ddiem.phenomebrowser.net/, accessed on 19 January 2025.

## Abbreviations

The following abbreviations are used in this manuscript:

IEM	inborn error of metabolism
CKD	chronic kidney disease
DDIEM	Drug Database for Inborn Errors of Metabolism
MMADHC	methylmalonic aciduria and homocystinuria
APRT	adenine phosphoribosyltransferase
GFR	glomerular filtration rate
UTI	urinary tract infection
AAV	adeno-associated virus
CAH	congenital adrenal hyperplasia
GRACILE	growth retardation, aminoaciduria, cholestasis, iron overload, lactic acidosis, early death
GSD	glycogen storage disease
HUS	hemolytic uremic syndrome
